# Knowledge of politician stock trading reduces congressional legitimacy and compliance with the law

**DOI:** 10.1073/pnas.2501822122

**Published:** 2025-05-20

**Authors:** Raihan Alam, Tage S. Rai

**Affiliations:** ^a^Rady School of Management, University of California, San Diego, CA 92093

**Keywords:** legitimacy, corruption, compliance, government

## Abstract

Institutional legitimacy is essential for democracies, yet public trust and confidence in the United States Congress are at an all-time low. A significant predictor of attitudes toward Congress is perceptions of corruption, with perceptions of corruption in government linked to less legitimacy. This study tests whether knowledge of Congressional stock trading affects legitimacy and compliance with Congressional authority. In a preregistered experiment with US citizens (*n* = 506), participants who read a report detailing how Congressmembers made higher-than-expected profits from stock trading in 2024 reported increased perceptions of corruption and decreased legitimacy in Congress. They also viewed laws passed by Congress as less fair and were less willing to comply with such laws. Perceptions of Congressional legitimacy mediated the effect of stock trading knowledge on willingness to comply with congressional laws and perceptions of congressional laws as fair. A preregistered follow-up experiment (*n* = 664) shows that these effects are not driven by how much Congressmembers profit but by how trading negatively affects broader perceptions of legitimacy. These findings highlight the detrimental effects of Congressional stock trading on perceptions of legitimacy and respect for the law.

Institutional legitimacy is essential for democratic functioning, fostering compliance with laws and cooperation with authorities even in the absence of rewards or threats of punishment (1). However, public trust in the US Congress has sharply declined: In 1958, 78% of Americans reported trusting the federal government to do the right thing almost always or most of the time (2). By April 2024, this figure had plummeted to just 22%, with most Democrats and Republicans expressing distrust in Congress. This erosion of trust has profound implications for democratic governance, as perceptions of legitimacy are linked to compliance with authorities, civic engagement, and less crime (3–6). One key predictor of trust and legitimacy is perceptions of corruption (7), which undermine beliefs that authorities act in the public interest. Scandals involving self-serving behavior by elected officials, such as the House Banking and Post Office scandals of the 1990s, have preceded declines in Congressional approval (8). More recently, Congressional stock trading has drawn public scrutiny as some members earn substantial financial gains through trades in industries they oversee legislatively (9), particularly amid allegations of trading shortly before the Trump administration’s tariff pause. These behaviors have sparked widespread criticism and renewed concerns about Congressional ethics.

This study examines how knowledge of Congressional stock trading affects public perceptions of trust, legitimacy, and compliance with Congressional authority. Drawing on studies of procedural justice (10–11), we propose that knowledge of Congressional stock trading erodes trust and increases perceptions of corruption in Congress, ultimately diminishing its legitimacy. Attenuated perceptions of legitimacy, in turn, are hypothesized to decrease compliance with Congressional laws and perceptions of congressional laws as fair. To test these claims, we conducted a preregistered survey experiment in January 2025, during the week after the Presidential inauguration, with 506 US citizens recruited online. Participants were randomly assigned to one of two conditions. In the trading condition, participants read a nonpartisan report (12) detailing how Congressmembers made significant profits from stock trading in 2024, significantly outperforming the S&P 500. In the control condition, participants read a report describing the educational backgrounds of Congressmembers. Both reports were similar in length and included graphs to support their information. After reading the reports, participants rated their trust in Congress, perceptions of corruption, and perceptions of legitimacy. They also evaluated the extent to which they viewed Congressional laws as self-serving, the extent to which they saw such laws as fair, and their willingness to comply with them. To address the limitation that the observed effects in Experiment 1 could be driven by financial outcomes themselves rather than perceptions of legitimacy, we conducted a preregistered follow-up experiment with 664 participants. Participants were randomly assigned to one of three conditions: profit, loss, or control. In the profit and loss conditions, we described a fictional Congressman who either made significant profits or incurred significant losses from stock trading in industries he legislates. The control condition described the Congressman’s legislative work with no mention of trading.

## Results

Experiment 1: We computed a one-way multivariate analysis of variance (MANOVA) (trading vs. control) and found a moderate effect of condition on perceptions of Congress *F* (6, 499) = 7.36, *P* < 0.001; η^2^ = 0.08. Post hoc analyses (Table 1) found that participants in the trading condition perceived Congress as significantly less trustworthy (*P* < 0.001), more corrupt (*P* < 0.001), and less legitimate (*P* < 0.001) than those in the control condition. They also viewed laws passed by Congress as less fair (*P* = 0.001) and more self-serving (*P* < 0.001) and were less willing to comply with these laws (*P* = 0.02) than participants in the control condition. Supplemental studies confirmed that the trading condition reduced perceptions of procedural fairness in Congress and that people view stock trading by Congressmembers in industries they legislate as unfair (*SI Appendix*). We tested the indirect effect (13) of knowledge of stock trading on legitimacy through a procedural justice composite measure of trust and corruption and found a significant indirect effect (*ab* = −0.44, *BCa CI* [−0.61, −0.28]) such that the trading condition reduced legitimacy by decreasing perceptions of procedural justice.

**Table 1.** Perceptions of Congress by Conditions from Experiment 1

Variable	*M_Trading_*(*SE*)	*M_Control_*(*SE*)	*f-*value	η^2^	*P* value
Trust	2.42(.078)	3.05 (0.078)	32.43	0.06	<0.001
Corruption	5.32(.084)	4.73 (0.085)	24.632	0.047	<0.001
Legitimacy	3.36(.093)	4.13 (0.094)	33.902	0.063	<0.001
Self-serving	5.26(.082)	4.89 (0.082)	10.441	0.02	<0.001
Fairness	3.35(.076)	3.79 (0.076)	17.134	0.033	0.001
Compliance	4.93(.084)	5.21 (0.085)	5.48	0.011	0.02

*Note. Error df* = 504

**Fig. 1.**
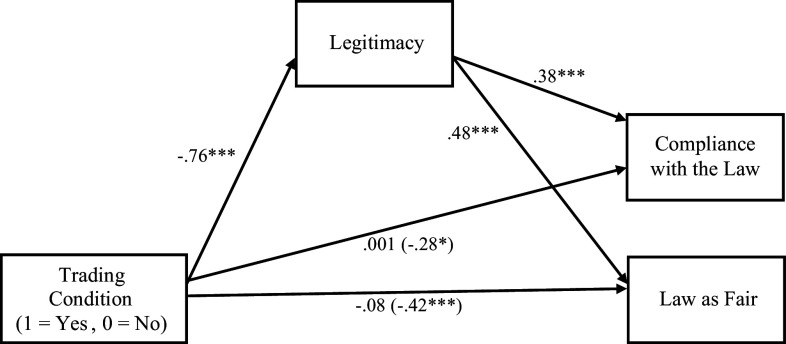
Effect of Trading Condition on Compliance and Perceiving Congressional Laws as Fair through Legitimacy from Experiment 1. ***P < 0.001, *P < 0.05.

Next, we tested the indirect effect of knowledge of stock trading on compliance with the law and perceptions of laws passed by Congress as fair through perceptions of legitimacy (Fig. 1). Results revealed a significant indirect effect of the trading condition on compliance through legitimacy (*ab* = −0.29, *BCa CI* [−0.41, −0.17]). The decrease in compliance in the trading condition was fully mediated by weaker perceptions of legitimacy, as the direct effect of the trading condition on compliance became nonsignificant when accounting for legitimacy (*P* = 0.99). The same pattern was found for law fairness (*ab* = −0.36, *BCa CI* [−0.49, −0.23]). The decrease in perceptions of fairness in the trading condition was fully mediated by weaker perceptions of legitimacy, as the direct effect of the trading condition on fairness became nonsignificant when accounting for legitimacy (*P* = 0.39). All effects persisted when controlling for political ideology.

**Fig. 2.**
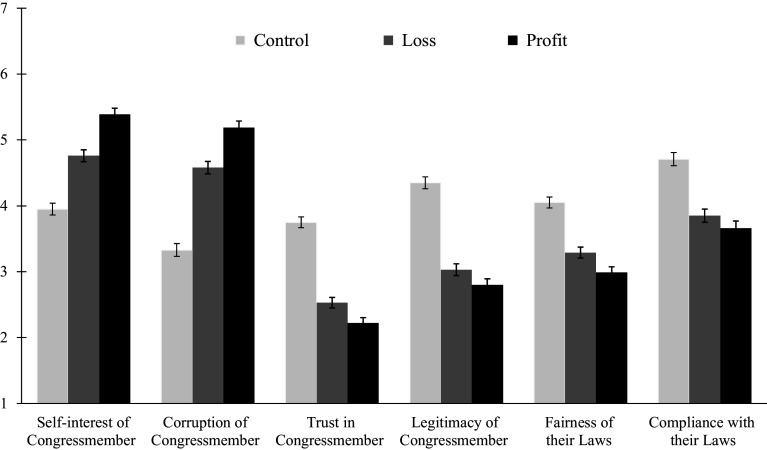
Perceptions of the Congressmember by Conditions from Experiment 2. Error bars represent SE. Image credit: UnusualWhales.

Experiment 2: We computed a one-way MANOVA and found a large effect of condition on perceptions of the Congressman *F* (12, 1312) = 22.26, *P* < 0.001; η^2^ =0.17 (Fig. 2 and Table 2). Post hoc analyses revealed significant differences across all dependent variables when comparing the control condition to both the loss and profit conditions (Table 2). Compared to the control condition, participants in the profit and loss conditions perceived the Congressman as less trustworthy (profit: *P* < 0.001, loss: *P* < 0.001), more corrupt (profit: *P* < 0.001, loss: *P* < 0.001), more self-serving (profit: *P* < 0.001, loss: *P* < 0.001), less legitimate (profit: *P* < 0.001, loss: *P* < 0.001), and rated his laws as less fair (profit: *P* < 0.001, loss: *P* < 0.001) and were less willing to comply with them (profit: *P* < 0.001, loss: *P* < 0.001). These effects were more pronounced in the profit condition for all variables (*P* < 0.02) except for legitimacy (*P* = 0.07) and compliance (*P* = 0.21).

**Table 2.** Perceptions of Congressmember by Conditions from Experiment 2

Variable	*M_Profit_*(*SE*)	*M_Loss_*(*SE*)	*M_Control_*(*SE*)	*f-*value	η^2^	*P* value Profit vs. Control	*P* value Loss vs. Control	*P* value Profit vs. Loss
Trust	2.22(.083)	2.53 (0.08)	3.75(.083)	100.408	0.223	<0.001	<0.001	0.01
Corruption	5.19(.098)	4.58 (0.094)	3.33(.097)	95.262	0.224	<0.001	<0.001	<0.001
Legitimacy	2.80(.092)	3.03 (0.09)	4.35(.09)	85.40	0.205	<0.001	<0.001	0.071
Self-serving	5.39(.092)	4.76 (0.09)	3.95(.09)	62.375	0.159	<0.001	<0.001	<0.001
Fairness	2.99(.085)	3.29 (0.083)	4.05(.083)	42.964	0.115	<0.001	<0.001	0.013
Compliance	3.66(.11)	3.85 (0.10)	4.71(.10)	29.437	0.082	<0.001	<0.001	0.211

*Note. Error df* = 661

Next, we tested the indirect effect of knowledge of stock trading on compliance with the Congressman’s laws and perceptions of his laws as fair through perceptions of his legitimacy. Given that both the profit and loss conditions differed in the expected directions from the control condition for all variables, we combined the two conditions into a single group and compared it to the control condition (*SI Appendix*, *Separate Mediation Models*). Results showed that there was an indirect effect of trading on compliance through legitimacy (*ab* = −0.81, *BCa CI* [−0.99, −0.65]). The decrease in compliance was fully mediated by weaker perceptions of the Congressman’s legitimacy, as the direct effect of knowledge of his stock trading on compliance became nonsignificant when accounting for legitimacy (*P* = 0.22). The same pattern was found for fairness of laws (*ab* = −0.78, *BCa CI* [−0.96, −0.63]). The decrease in perceptions of fairness was fully mediated by weaker perceptions of the Congressman’s legitimacy, as the direct effect of knowledge of his stock trading on fairness became nonsignificant when accounting for legitimacy (*P* = 0.13).

## Discussion

These findings highlight the detrimental effects of Congressional stock trading on public perceptions of institutional legitimacy. Knowledge of Congressional stock trading significantly reduced trust, increased perceptions of corruption, and undermined perceived fairness of laws. Critically, diminished perceptions of legitimacy mediated the effects of stock trading knowledge on compliance and fairness judgments—effects that cannot be solely attributed to beneficial financial outcomes for Congressmembers.

These results suggest that policies aimed at curbing insider trading by Congressmembers, which have bipartisan political support (14), could mitigate public perceptions of corruption and improve Congressional legitimacy. One limitation of our study is that it only measured the immediate effects of stock trading knowledge. While prior research shows that corruption can have lasting effects on legitimacy (8, 15), future research should examine whether these specific effects persist and how repeated exposure to corruption accumulates over time. Longitudinal studies or natural experiments leveraging future real-world legislative reforms on Congressional stock trading could provide insight into the durability of these perceptions.

## Materials and Methods

In January 2025, we recruited participants (*n* = 506 for Experiment 1, *n* = 664 for Experiment 2) through CloudResearch for an online survey and paid them $0.25. Participants were randomly assigned to conditions. After the manipulation, participants in Experiment 1 rated their trust in Congress, perceptions of corruption and legitimacy, views of congressional laws, and willingness to comply with congressional laws. In Experiment 2, they made the same ratings about a fictional congressman. See *SI Appendix* for full stimuli and survey items. Research was approved by the University of California, San Diego Institutional Review Board. All participants gave informed consent.

## Supplementary Material

Appendix 01 (PDF)

## Data Availability

Preregistrations and data are available at OSF (https://osf.io/xw7p2/?view_only=512743e601db405ca8160cefc2d25c50) (16). All other data are included in the manuscript and/or *SI Appendix*.
