# Supplementary material for: Knowledge of politician stock trading reduces congressional legitimacy and compliance with the law
Source: Proc Natl Acad Sci U S A. 2025 May 20;122(21):e2501822122. doi: 10.1073/pnas.2501822122 (PMC12130894; doi:10.1073/pnas.2501822122)
Supplement: Supplementary file 1 — Appendix 01 (PDF) [file pnas.2501822122.sapp.pdf]

Supplemental Information
Manipulations and Measures for Each Experiment

EXPERIMENT 1

Control Condition

A recent report from Statista revealed data about the educational backgrounds of U.S. Congress members. The report highlighted the colleges and universities where members of the 117th Congress received their first degree. While prestigious schools like Harvard University were common, with 20 members graduating from there, a variety of public and private institutions were represented as well.

The University of California and Stanford University featured prominently, with 17 and 14 members, respectively, receiving their undergraduate degrees from these institutions. Other schools, such as the University of Wisconsin and Georgetown University, were among the top institutions attended by Congress members. These universities are located across the country, reflecting the diverse educational pathways of U.S. lawmakers.

The graph below shows the number of Congress members from the 117th Congress who received their first degree from the top institutions. Blue bars represent Senators, while red bars represent Representatives. The data emphasizes the range of schools represented, showing that Congress members come from a wide variety of academic backgrounds.

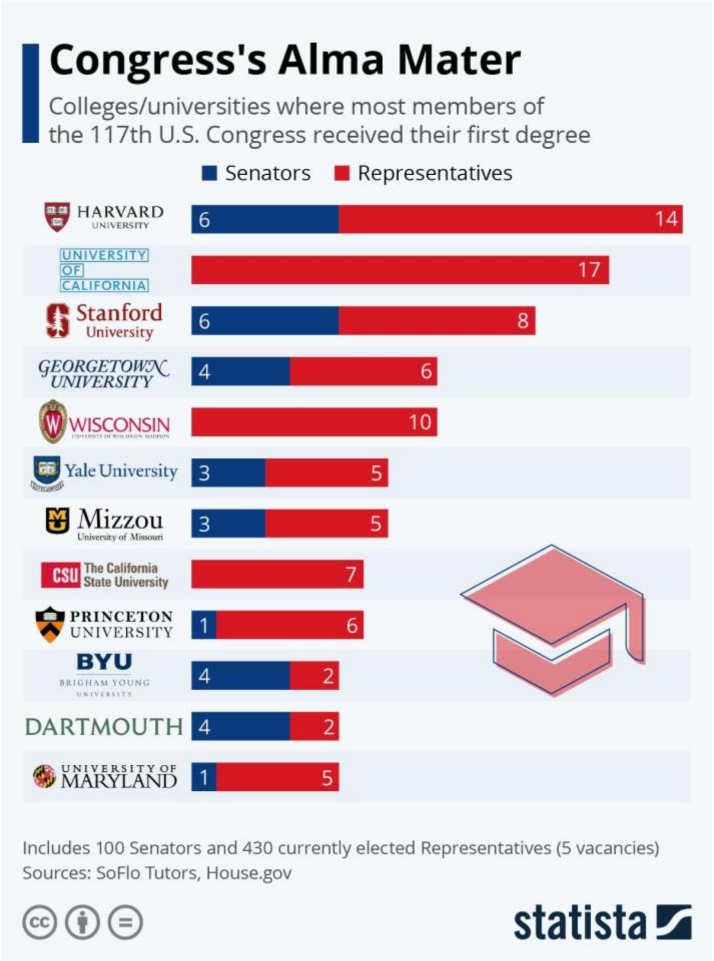

Stock Trading Condition

A recent report from Unusual Whales, a watchdog group, revealed striking patterns in the stock market performance of U.S. Congress members in 2024. According to the report, many Congress members significantly outperformed the S&P 500 (SPY), which gained approximately 10% over the same period. The S&P 500 is a stock market index that tracks the performance of 500 of the largest publicly traded companies in the United States, serving as a benchmark for overall market performance.

The report highlighted that several Congress members achieved portfolio gains exceeding 100%, with one member making an increase of nearly 150%. These gains were made in stocks across various industries, including technology, defense, and healthcare—sectors that Congress actively makes laws about.

The graph below illustrates the weighted percent change in Congressional stock portfolio values over 2024. The yellow line represents the S&P 500's (SPY) annual return of approximately 10%. Bars represent individual Congress members, with Democrats shown in blue and Republicans in red. The data highlights the substantial gains made by several members, far outpacing the market benchmark.

This information was made available under the STOCK Act, which requires members of

Congress to file Periodic Transaction Reports within 45 days of trading any stock. Stock trading by Congress members is not illegal under current U.S. law.

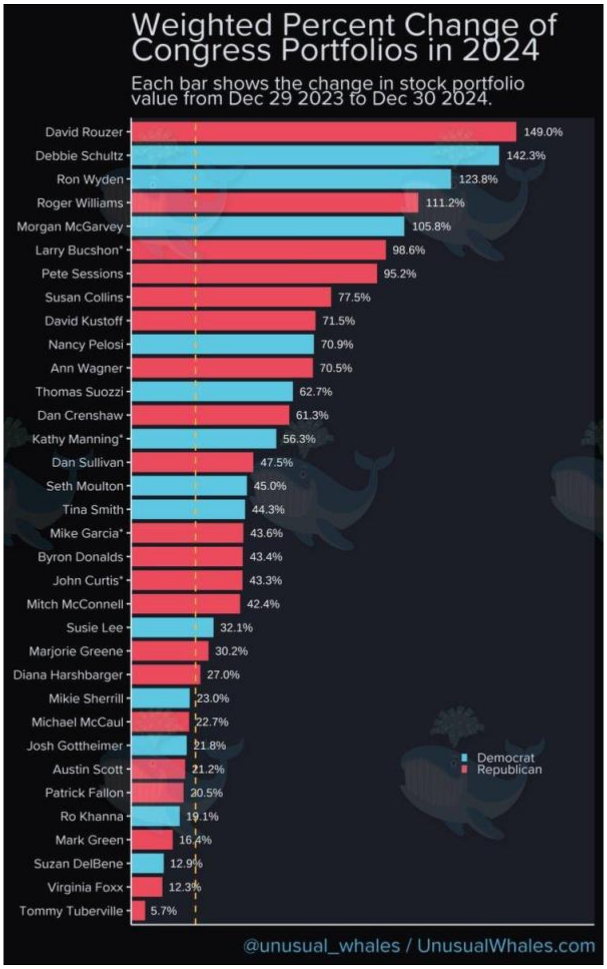

Image credit: UnusualWhales.

<u>EXPERIMENT 2</u>

<u>Control Condition</u>

Congressman Jonathan Brown has served in the House of Representatives since 2018. During his time in office, he has worked on a variety of legislative committees, including those focused on agriculture, infrastructure, and taxes.

<u>Losing Money Condition</u>

Congressman Jonathan Brown has lost significant money from stock trading while serving in the House of Representatives. For instance, in 2024, Congressman Brown lost over $1 million trading stocks in industries such as defense and healthcare—sectors that he has been involved in making laws and regulations. Critics have suggested that these trades may have been informed by his insider knowledge. This information was disclosed under the STOCK Act, which requires members of Congress to file Periodic Transaction Reports within 45 days of trading any stock.

Gaining Money Condition

Congressman Jonathan Brown has made significant profits from stock trading while serving in the House of Representatives. For instance, in 2024, Congressman Brown made over $1 million trading stocks in industries such as defense and healthcare—sectors that he has been involved in making laws and regulations. Critics have suggested that these trades may have been informed by his insider knowledge. This information was disclosed under the STOCK Act, which requires members of Congress to file Periodic Transaction Reports within 45 days of trading any stock.

Dependent Variables (all answered from 1, not at all, to 7, completely)

EXPERIMENT 1

How much do you trust Congress?

How corrupt do you think Congress is?

How legitimate do you believe Congress is as a governing body?

How fair do you think the laws passed by Congress are?

To what extent do you think the laws passed by Congress benefit Congress members themselves rather than the public?

How willing are you to comply with laws passed by Congress?

EXPERIMENT 2

How much do you trust Congressman Brown?

How corrupt do you think Congressman Brown is?

How legitimate do you believe Congressman Brown is as a lawmaker?

How fair do you think the laws passed by Congressman Brown are?

To what extent do you think the laws passed by Congressman Brown benefit himself rather than the public?

How willing are you to comply with laws passed by Congressman Brown?

Demographic Variables

Here is a 7-point scale on which the political views that people might hold are arranged from extremely liberal to extremely conservative.

- Extremely liberal
- Liberal
- Slightly Liberal
- Moderate: Middle of the Road
- Slightly conservative
- Conservative

- Extremely conservative

Please indicate your age:

__

What is your sex:

- Male
- Female
- Do not identify

How would you describe your class?

- Upper class
- Upper middle class
- Middle class
- Lower middle class
- Working class/lower class

What is your ethnicity?

- African-American/Black
- Asian-American/Asian
- European-American/White
- East Indian
- Hispanic/Chicano/Latino
- Native American
- Other (Please indicate) _______

What is the highest level of education you have completed?

- High school or less
- 2-year college (associate's degree)
- 4-year college (BA, BS)
- Masters degree (MA, MS)
- PhD, MD, JD, or other higher degree

Attention and Manipulation Checks

BOTH EXPERIMENTS

Select "2" if you are paying attention.

- 1
- 2
- 3
- 4
- 5

- 6
- 7

EXPERIMENT 1

What was the topic your read about?

- Whale migration
- Stock trading among members of Congress
- The educational background of members of Congress
- Homelessness

EXPERIMENT 2

What did you learn about Congressman Brown?

- He has lost money from trading stocks during his time in Congress
- He enjoys swimming in his free time
- He has worked on agriculture, infrastructure, and taxes during his time in Congress
- He has gained money from trading stocks during his time in Congress

Analyses and Experiments not Presented in the Main Text

Supplemental Experiment on Procedural Fairness

To confirm that knowledge of Congressional stock trading impacts procedural fairness perceptions, we recruited participants (*n* = 221) from Amazon's Mechanical Turk and paid them $.25 in an additional study. A sensitivity analysis indicated that our sample size would allow us to reliably detect an effect size of η^2 = .04 or greater with 80% power and alpha at .05. Participants were randomly assigned either the stock trading (*n* = 110) or control condition (*n* = 111) from Experiment 1. After exposure to condition, we asked participants about procedural fairness in Congress with the following item: "How fair do you think the procedures used by Congress to pass laws are" on a 1-7 scale (1 = not at all fair, 7 = completely). A between-subjects ANOVA revealed that participants in the stock trading condition viewed Congressional legislative procedures as significantly less fair (*M* = 3.24, *SE* = .12) compared to participants in the control condition (*M* = 3.95, *SE* = .12), $F(1, 219) = 17.84$, $p < .001$, $\eta^2 = .075$.

Supplemental Study on Perceptions of Stock Trading

To confirm that people view stock trading by members of Congress as unfair, we recruited participants (*n* = 99) from Amazon's Mechanical Turk and paid them $.25 in an additional study. We asked participants the following question: "How fair do you it is for Congress members to trade stocks in industries they make laws about" on a 1-7 scale (1 = not at all, 7 = completely). Descriptive statistics revealed that participants viewed stock trading by Congress members as unfair, with a mean score of 1.86 (*SD* = 1.48), well below the midpoint.

Experiment 2: Separate Mediation Models

Profit vs. Control: Results showed that there was indirect effect of the trading on compliance through legitimacy (*ab* = -.881, *BCa CI* [-1.10, -.666]). The decrease in compliance

was fully mediated by weaker perceptions of the Congressmember's legitimacy, as the direct effect of knowledge of his stock trading on compliance became non-significant when accounting for legitimacy (p = .20). For law fairness, results showed there was an indirect effect of the trading on perceptions of fairness through legitimacy (ab = -.797, *BCa CI* [-.981, -.622]). The decrease in perceptions of fairness was partially mediated by weaker perceptions of the Congressmember's legitimacy, as the direct effect of knowledge of his stock trading on fairness was still significant when accounting for legitimacy (p = .01).

Loss vs. Control: Results showed that there was indirect effect of the trading on compliance through legitimacy (ab = -.757, *BCa CI* [-.956, -.572]). The decrease in compliance was fully mediated by weaker perceptions of the Congressmember's legitimacy, as the direct effect of knowledge of his stock trading on compliance became non-significant when accounting for legitimacy (p = .39). For law fairness, results showed there was an indirect effect of the trading on perceptions of fairness through legitimacy (ab = -.690, *BCa CI* [-.862, -.528]). The decrease in perceptions of fairness was fully mediated by weaker perceptions of the Congressmember's legitimacy, as the direct effect of knowledge of his stock trading on fairness became non-significant when accounting for legitimacy (p = .42).

Information about the Mediation Models

For all mediation models, we used Model 4 of Hayes PROCESS (13) with 10,000 bias-corrected bootstrap resamples.
